# Light-Controllable PROTACs for Temporospatial Control of Protein Degradation

**DOI:** 10.3389/fcell.2021.678077

**Published:** 2021-07-19

**Authors:** Jing Liu, Yunhua Peng, Wenyi Wei

**Affiliations:** Department of Pathology, Beth Israel Deaconess Medical Center, Harvard Medical School, Boston, MA, United States

**Keywords:** ubiquitin, E3 ligase, PROTAC, tumorigenesis, light controllable

## Abstract

PROteolysis-TArgeting Chimeras (PROTACs) is an emerging and promising approach to target intracellular proteins for ubiquitination-mediated degradation, including those so-called undruggable protein targets, such as transcriptional factors and scaffold proteins. To date, plenty of PROTACs have been developed to degrade various disease-relevant proteins, such as estrogen receptor (ER), androgen receptor (AR), RTK, and CDKs. However, the on-target off-tissue and off-target effect is one of the major limitation that prevents the usage of PROTACs in clinic. To this end, we and several other groups have recently developed light-controllable PROTACs, as the representative for the third generation controllable PROTACs, by using either photo-caging or photo-switch approaches. In this review, we summarize the emerging light-controllable PROTACs and the prospective for other potential ways to achieve temporospatial control of PROTACs.

## Introduction

**FIGURE 1:**
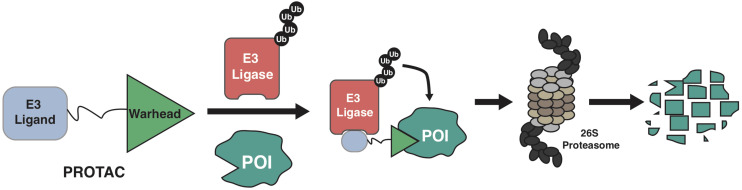
A schematic diagram for the action model of PROTAC. PROTAC recruits endogenous E3 ligase to ubiquitinate protein of interests (POIs), thus promoting the subsequent degradation of POI by the 26S proteasome.

The ubiquitin-proteasome system (UPS) governs the degradation and turnover of protein, thus playing critical functions in many cellular processes including protein quality control, cell cycle progression, and cell signaling transduction (Komander and Rape, 2012; Pohl and Dikic, 2019). Catalyzed by the ubiquitin-activating enzyme (E1), the ubiquitin is transferred onto the ubiquitin-conjugating enzyme (E2), and eventually transferred onto protein target by the E3 ubiquitin ligase. The selectivity of the ubiquitination process on a protein substrate primarily relies on its recognition by a E3 ubiquitin ligase (Pickart, 2001; Bernassola et al., 2008; Zhou et al., 2013), through a short sequence motif on the protein substrate, known as degron (Mészáros et al., 2017; Kumar et al., 2020). For instance, the SCF^β–TrCP^ E3 ligase recognizes the phospho-degron of DpSGXXpS/pT (X represents any amino acid, and p represents phosphorylation modification), and the von Hippel-Lindau (VHL) E3 ligase binds to substrates with the proline-hydroxyl-degron of LAP-OH (P-OH represents the hydroxylation on the proline). Based on the growing understanding about biological function of E3 ligase and UPS, PROteolysis TArgeting Chimera (PROTAC) emerges as a new pharmaceutical approach since 2001 (Sakamoto et al., 2001). By hijacking the endogenous UPS to specifically degrade proteins of interest (POI), PROTACs are theoretically capable of targeting any proteins in cells (Sakamoto, 2010; Neklesa et al., 2017; Churcher, 2018; Guo et al., 2019; Paiva and Crews, 2019). Of the three functional moieties in the PROTAC molecule, the E3 ligase-ligand is designed for recruiting endogenous E3 ubiquitin ligase, and the warhead part (or called target-recruiting ligand) determines the specificity of protein targets, while the linker region between them should be optimized to achieve best efficiency and specificity to degrade individual substrate, in a case-by-case manner (Figure 1; Flanagan and Neklesa, 2019; Pettersson and Crews, 2019).

The first generation of PROTACs take advantage of degron-derived peptides, such as phospho-peptides (Sakamoto et al., 2001, 2003) or hydroxyl-peptides (Schneekloth et al., 2004; Zhang et al., 2004; Rodriguez-Gonzalez et al., 2008), to recruit the endogenous β-TrCP or VHL E3 ubiquitin ligases, respectively. These peptide-based PROTACs have relatively high molecule weight, which limits their permeability into cells and their function as a *bona fide* drug. Moreover, peptide is unstable, and could only be injected into target cells, making them not practical in clinic. Recently, a modified version of peptide-based PROTAC, TD-PROTAC (Jiang et al., 2018), has been developed with better stability and cell-permeability, making it capable of degrading ERα *in vitro* and *in vivo*.

Besides these degron-derived peptides, small molecule inhibitors or binding partners have been developed for several E3 ligase, such as auxin for TIR E3 ligase (Dharmasiri et al., 2005), nutlin for mouse double minute 2 homolog (MDM2) E3 ligase (Vassilev et al., 2004). Based on these specific binding ligands of E3 ligases, the second generation small molecule PROTACs have been developed. In 2008, the first nutlin-based small molecule PROTAC has been developed to target androgen receptor (AR) for degradation in prostate cancer cells (Schneekloth et al., 2008). A recent study has shown that compared with VHL-based PROTACs, MDM2-based PROTACs might offer a synergistic anti-proliferative activity to cancer cells (Hines et al., 2019), in part due to the degradation of target protein bromodomain-containing protein 4 (BRD4), as well as the stabilization and accumulation of the tumor suppressor p53, a well-characterized endo-substrate of MDM2 (Chene, 2003). Several antagonists of cellular inhibitor of apoptosis protein 1 (cIAP1) E3 ligase, including bestatin (Sato et al., 2008), methyl bestatin (MeBS) (Sekine et al., 2008), MV1 (Varfolomeev et al., 2007) and LCL161 (Yang et al., 2016) have been reported to bind with cIAP1 and to promote its auto-ubiquitination and degradation. These small molecule antagonists have also been used in targeted protein degradation (TPD), also known as Specific and Non-genetic IAP-dependent Protein ERaser (SNIPER), to degrade many protein targets such as AR (Shibata et al., 2018), BCL-ABL (Demizu et al., 2016; Shibata et al., 2017; Shimokawa et al., 2017), BRDs (Ohoka et al., 2017b, 2019), Bruton’s tyrosine kinase (BTK) (Tinworth et al., 2019), cellular retinoic acid-binding protein 2 (CRABP2) (Okuhira et al., 2017), estrogen receptor (ER) (Okuhira et al., 2013), and transforming acidic coiled-coil containing protein 3 (TACC3) (Ohoka et al., 2014, 2017a).

In 2010, pomalidomide and its analogs immunomodulatory imide drugs (IMiDs) have been defined as molecule glues to bind with the endogenous cereblon (CRBN) E3 ligase (Ito et al., 2010; Fischer et al., 2014), subsequently causing the proteasomal degradation of several neo-substrates, including IKZFs (Kronke et al., 2014; Lu et al., 2014), CK1α (Kronke et al., 2015), GSPT1 (Matyskiela et al., 2016), SALL4 (Donovan et al., 2018), p63 (Asatsuma-Okumura et al., 2019) and ARID2 (Yamamoto et al., 2020). In 2015, IMiDs as ligands of the CRBN E3 ligase have been firstly used to develop CRBN-based PROTACs for the degradation of BRD4 and FKBP12 (Winter et al., 2015), and to date CRBN-based PROTACs have been applied to more than 30 different protein targets, for the treatment of cancer and inflammation disease (Supplementary Table 1; Mullard, 2021), among which ARV-110 (Neklesa et al., 2018) (NCT03888612) and ARV471 (Flanagan et al., 2019) (NCT04072952) are in Phase I/II clinical trials for the treatment of prostate cancer (Petrylak et al., 2020) and breast cancer (BRCA), respectively. In 2012, the small molecule VHL ligand (VHL ligand 1) has been developed to specifically interact with VHL without an inhibitory effect to the tumor suppressive function of the VHL E3 ligase (Buckley et al., 2012a,b; Galdeano et al., 2014). Furthermore, several other modified VHL ligands have been developed, including the 1, 3-fluoro-4-hydroxyprolines and methyl-VHL ligand 1 (Testa et al., 2018). Using these small molecule VHL ligands, dozens small molecule VHL-based PROTACs have been developed to target intracellular proteins, including AR (Salami et al., 2018; Han et al., 2019) and ER (Hu et al., 2019; Kargbo, 2019; Supplementary Table 1).

Compared with small molecule inhibitors, PROTACs have several advantages. First, unlike typical reversible enzymatic inhibitors, active center or allosteric site of protein targets is not necessary for PROTACs, making it possible to target those so-called undruggable proteins. Second, PROTACs function in a catalytic manner, and the drug could be recycled after the protein target being degraded, making it more potent than small molecule inhibitors. However, the catalytic feature of PROTACs might also introduce potential higher toxicity to cells in part due to the off-tissue on-target effects and off-target effects (Raina et al., 2016; Moreau et al., 2020), which is one of the major limitation for their application in practice. For example, thalidomide has been approved in 1950s for treating morning sickness in pregnant women in Europe, which caused a tragedy that affects thousands of children with severe birth defects (Rehman et al., 2011). Until recent, the teratogenic effects is defined for CRBN-mediated degradation of p63 (Asatsuma-Okumura et al., 2019) and SALL4 (Donovan et al., 2018). Besides, more and more CRBN neo-substrates of IMiDs have been reported, including IKZFs (Kronke et al., 2014; Lu et al., 2014), CK1α (Kronke et al., 2015), GSPT1 (Matyskiela et al., 2016), ARID2 (Yamamoto et al., 2020), RNF166 (You et al., 2020). ZNF827, and ZFP91 (Zorba et al., 2018). Furthermore, the subcutaneous injection of BRD4 degrader ARV-771 in xenograft tumor mice causes noticeable skin discoloration (Raina et al., 2016), which is consistent with the phenotype of *Brd4* depleted mice (Bolden et al., 2014). Thus, next generation of PROTACs should at least have the property to distinguish target versus non-target tissues/cells to alleviate its toxicity issue.

## The Third Generation PROTACs With Targeting Delivery And/Or Controllable Activation

One way to achieve targeted degradation of protein is to specifically deliver PROTACs into cancer cells, by taking advantage of the receptors expressed on the membrane of cancer cells, but not of normal cells. Recently, the antibody drug-conjugate (ADC) approach has been adopted for delivering PROTACs into cancer cells that expressing cancer-specific membrane-anchored receptors, such as HER2 (Dragovich et al., 2020, 2021a,b; Maneiro et al., 2020; Pillow et al., 2020). A major disadvantage of antibody-conjugated PROTAC is its relatively high molecule weight and weak stability. Thus, we have recently developed a small molecule version of targeting delivery platform for PROTACs, namely folate-PROTAC (Liu et al., 2021), by conjugating a folate group on the hydroxyl group of VHL ligand, to specific deliver PROTACs into cancer cells that express relatively high levels of folate receptor α (FOLR1) (Scaranti et al., 2020). Moreover, PROTACs that recruits cancer-specific E3 ligase might provide a way to achieve cancer-selective action of PROTACs (Nalawansha and Crews, 2020). For example, VHL-based PROTAC for BCL-xl is more tolerable than BCL-xl inhibitor ATB263, in part due to the relatively low expression of VHL in platelets than in cancer cells, thus reducing potential on-target toxicity (Khan et al., 2019). Several cancer specific or tissue specific E3 ligases have been recently identified (Schapira et al., 2019), however, none of these E3 ligase has ready-to-use small molecule binders yet, which prevents its further clinical development.

Another approach to achieve controllable protein degradation is to use an extraneous cellular signaling for the activation of PROTAC, such as by phosphoPROTACs (Hines et al., 2013). After stimulated with either nerve growth factor (NGF) or neuregulin, the two prototype phosphoPROTACs degraded fibroblast growth factor receptor substrate 2α (FRS2α) or PI3K, respectively (Hines et al., 2013). The phosphoPROTACs provide an option for controllable-PROTACs, but it still lacks tissue/cell specificity as these extraneous cues largely rely on universal receptors that are expressed in all cells regardless of normal or tumor cells. Recently, we and several other laboratories have independently developed light-controllable PROTACs, using either photo-cage or photo-switch approaches, which are widely used in photodynamic therapy (PDT) (Bethea et al., 1999; Moore et al., 2009; Agostinis et al., 2011; Shafirstein et al., 2016). Here, we summarize these light-controllable PROTACs and discuss for the advantages and limits for their applications in clinic.

## Photo-Cage Enables Controllable PROTAC Activation to Degrade Proteins in Targeting Cells

### Photo-Cage and Photo-Cage Chemical Group

**FIGURE 2:**
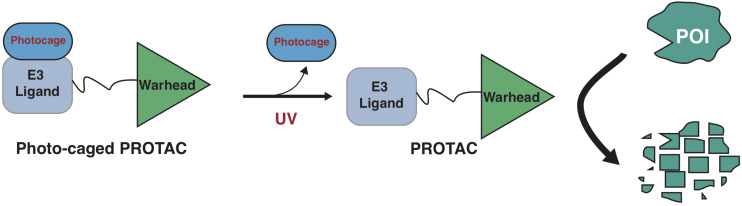
A schematic diagram for action model of photo-caged-PROTAC under control of UV illumination. The photo-caged-PROTAC is inert at beginning and activated by UV illumination, which leads to the release of the photocage group, thus enabling the degradation of POI in a controllable manner.

Photo-cage groups, also known as photoremovable protecting groups, provide a standard approach to spatially and temporally control the release of chemicals in cells. To date, several types of photo-cage groups have been develop for the purpose of controlled release of organic molecules (Klan et al., 2013). However, only a few types of photo-cage groups are available for caging small molecule drugs, in part due to the strict release condition in water solution rather than other organic solution, such as methanol or ethanol (Klan et al., 2013). In the past few years, the development in biorthogonal chemistry prompts several photolabile groups for caging cellular molecules such as neurotransmitters, secondary messengers, and amino acids (Bardhan and Deiters, 2019), making it a powerful tool in biological studies. Taking advantage of these photo-cage groups, we and other groups have recently developed photo-caged PROTACs, which enable controllable activation of PROTACs in target cells (Xue et al., 2019; Liu et al., 2020; Naro et al., 2020; Figure 2).

### Photo-Cage Approach for CRBN-Based PROTACs

**FIGURE 3:**
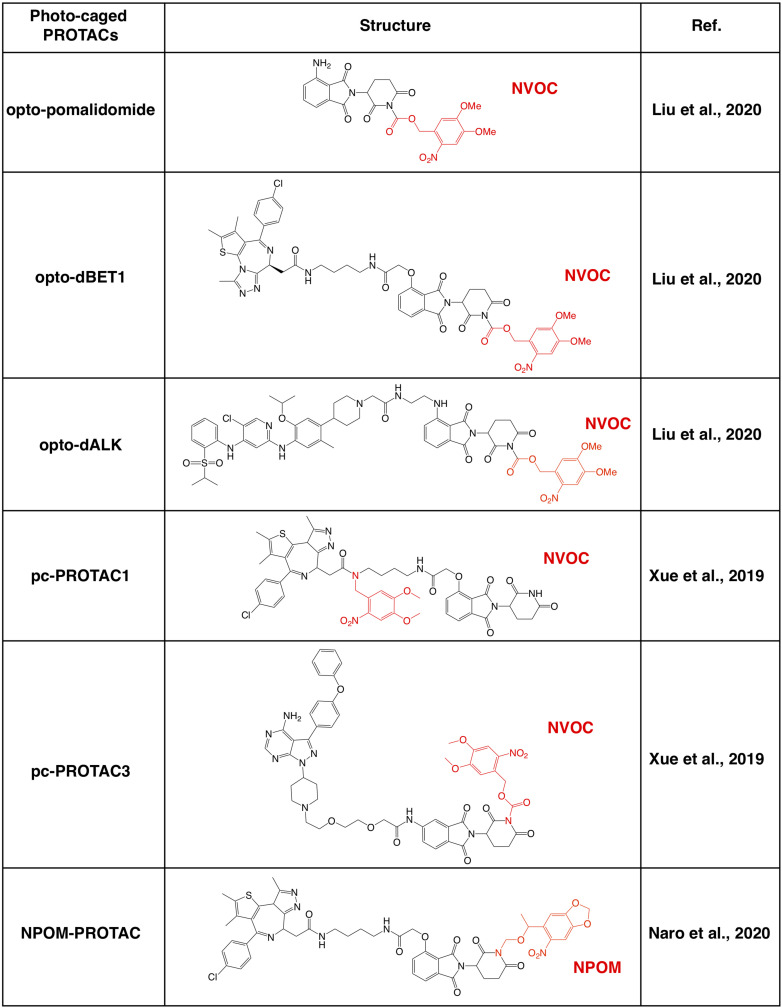
Summary of photo-caged CRBN-based PROTACs. The photo-cage groups are marked in red. NVOC, nitroveratryloxycarbonyl; NPOM, 6-nitropiperonyloxymethyl.

Further investigations on the crystal structure of CRBN and phthalimide complex indicate that the glutarimide NH in phthalidomide is critical for its binding with CRBN, particularly for the backbone carbonyl of the His380 residual (Petzold et al., 2016; Sievers et al., 2018; Matyskiela et al., 2020). Caging of glutarimide NH with methyl or other groups completely abolish the ability of pomalidomide to bind with the CRBN E3 ligase, and methyl-PROTACs are usually used as negative controls during the designation of PROTACs (Bondeson et al., 2018; Zhang et al., 2018). There are several photo-caged CRBN-based PROTACs that have been reported, including opto-PROTAC (Liu et al., 2020), pc-PROTAC (Xue et al., 2019), and others (Naro et al., 2020; Figure 3).

By incorporated a reversible photo-cage group, nitroveratryloxycarbonyl (NVOC) on the glutarimide NH of pomalidomide, opto-pomalidomide is inert and loss the capability in degrading IKZFs in cells (Liu et al., 2020), thus might be suitable to be applied to any other CRBN-based PROTACs. Two prototype opto-PROTACs, opto-dBET1 and opto-dALK, are inert and could be activated only after illuminated with UVA (λ = 365 nm) to degrade BRDs and ALK-fusion proteins, respectively (Liu et al., 2020). From another independent report, by using a similar photo-cage approach with NVOC, two pc-PROTACs prototypes, pc-PROTAC1 and pc-PROTAC3, degrade BRD4 and BTK, respectively, only after UVA illumination (Xue et al., 2019). Furthermore, by using a zebrafish model, they have validated the capability of pc-PROTAC1 in degrading endogenous BRDs under the control of UVA (λ = 365 nm) *in vivo* (Xue et al., 2019). Moreover, another photo-cage group, 6-nitropiperonyloxymethyl (NPOM) has also been used to cage the glutarimide NH in dBET1, and the resulting photo-caged PROTAC could degrade BRD4 after being illuminated with UVA (λ = 402 nm) (Naro et al., 2020). These studies together indicate that photo-cage on the glutarimide NH group could likely be an universal way for developing light-controllable PROTACs, and might be easily applied to other CRBN-based PROTACs in future studies.

### Photo-Cage Approaches for VHL-Based PROTACs

**FIGURE 4:**
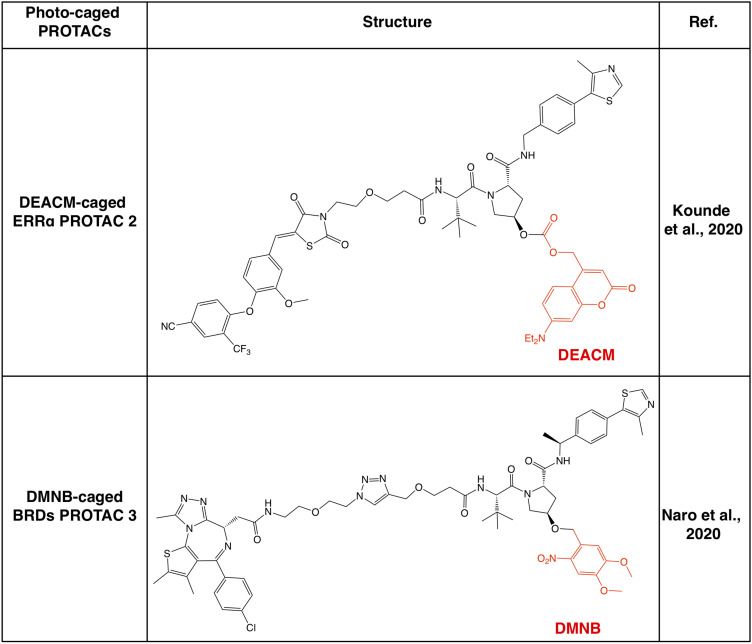
Summary of photo-caged VHL-based PROTACs. Photo-cage groups are marked in red. The photo-cage groups are in red. DMNB, 4,5-dimethoxy-2-nitrobenzyl; DEACM, diethylamino coumarin.

Apart from CRBN-based PROTACs, VHL-based PROTACs represent another major class of second-generation small molecule PROTACs, and the photo-cage approach has also been used in VHL-based PROTACs (Figure 4). In a recent study, a photocleavable 4,5-dimethoxy-2-nitrobenzyl (DMNB) group has been incorporated onto the hydroxyl group of VHL ligand 1, and a prototype caged-PROTAC could degrade BRD4 after irradiation with UVA (λ = 365 nm) (Kounde et al., 2020). In another independent study, the photo-cage group diethylamino coumarin (DEACM) has been used to cage the VHL ligand in VHL-based PROTAC against ERRα, and the resulting caged-PROTAC is inert and regains the ability to degrade ERRα after activated by UVA (λ = 360 nm) (Naro et al., 2020). Given that the incorporation of photo-cage groups only affects the binding between PROTACs and the VHL E3 ligase, but not the protein substrate, those reported photo-cage methods could also be applied to other VHL-based PROTACs.

## Photo-Switch Provide a Reversible On/Off Shift for PROTAC to Degrade Intracellular Proteins in Target Cells

### Photo-Switchable Chemical Group in Biology

**FIGURE 5:**
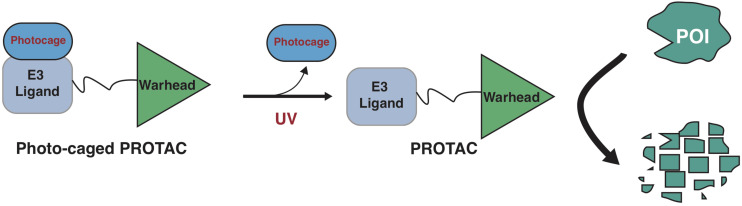
A schematic diagram for action model of photo-switch-PROTAC. The photo-switch PROTACs can be switched on and off by illumination with different wavelengths of light, which leads to the switch between the *cis* and *trans* forms of the photo-switch-PROTAC.

**FIGURE 6:**
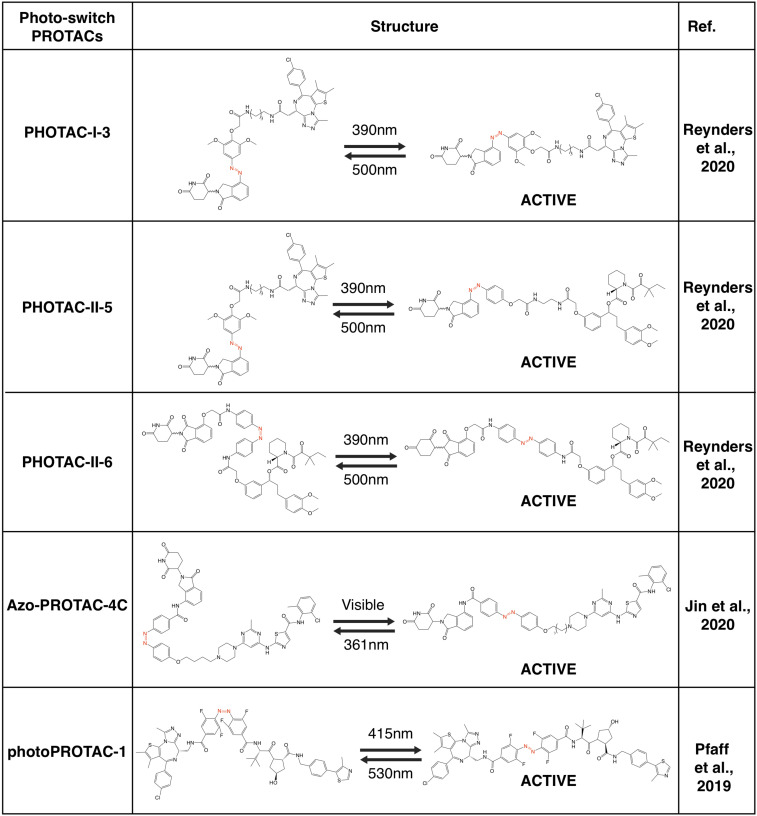
Summary of photo-switch PROTACs.

After entering target tissues/cells, focal UVA illumination leads to the release of activated PROTACs to be functional (Xue et al., 2019; Kounde et al., 2020; Liu et al., 2020; Naro et al., 2020). Activated PROTACs constantly degrade protein targets, and the degradation process will not stop before the clearance of PROTAC molecules. Thus, theoretically it should be better to add another OFF switch to inactivate the PROTACs, and the photo-switch provides a practical way. To this end, by taking advantage of the light-switchable azobenzene group or its analogs, several photo-switch PROTACs have been developed, including PHOTACs (Reynders et al., 2020), Azo-PROTACs (Jin et al., 2020) and photoPROTACs (Pfaff et al., 2019; Figures 5, 6).

### Photo-Switch PROTACs

Recently, several groups have utilized the photoswitch approach, i.e., azobenzene, to achieve photochemical isomerization of PROTAC molecules, and those photo-switch PROTACs could be reversibly turned on and off with light of different wave lengths (Reynders et al., 2020). By incorporating an azobenzene group in the linker region of pomalidomide-derived PROTACs, a type of light-inducible PROTACs, namely PHOTACs have developed. The two prototype PHOTACs remain in a *trans* inactive form in visible light (λ = 525 nm), and could be switched on with UVA illumination (λ = 390 nm), which leads to the conformation change to a *cis* active form, thus becoming capable of degrading BRDs and FKBP12, respectively. Furthermore, these PHOTACs could be turned off by visible light (λ = 525 nm), where PHOTACs return to the *trans* inactive form (Reynders et al., 2020). Furthermore, a similar photoswitchable azobenzene-based approach has been adopted in CRBN-based PROTACs to develop Azo-PROTACs. The prototype Azo-PROTAC could be switch between the *trans* active (ON) and the *cis* inactive (OFF) forms with either visible light or UV-C illumination, to ensure the light-controlled degradation of BCR-ABL fusion and ABL proteins in myelogenous leukemia K562 cells (Jin et al., 2020). Similarly, photo-switch could also be applied to VHL-based PROTAC. In another independent study, by using a similar photo-switch method to VHL-based PROTAC, photoPROTACs adopt the ortho-F4-azobenzene in the linker region between VHL ligand and warhead moiety against protein target (Pfaff et al., 2019). In contrast with PHOTACs, photoPROTACs remains as *cis* inactive form at beginning, and could be activated by UVA (λ = 415 nm) to change into a *trans* active form. Further illuminated by visible light (λ = 530 nm) could turn off the photoPROTAC, and the prototype photoPROTAC-1 could be switched on and off to degrade BRDs in cells in a light-controllable manner (Pfaff et al., 2019).

## Limitations of Light-Controllable PROTACs and Perspective

The potential on-target off-tissue effects and off-target effects limit the application of PROTACs in clinic. These third-generation controllable PROTACs using light to activate or inactivate the PROTAC provide another layer of regulation on PROTACs, making it more practicable and controllable. However, those light-controllable PROTACs also have some disadvantages.

Notably, UVA light is used to activate or inactivate these light-controllable PROTACs, however, UVA light might trigger damage to DNA (Mouret et al., 2006; Cadet and Douki, 2011), especially when used in patients. Compared with UVB with shorter wavelength that causes DNA damage by triggering pyrimidine dimerization, UVA is less genotoxic (de Gruijl, 2002). However, UVA radiation is still thought to induce oxidant stress and DNA damage, which causes skin aging and possible skin cancers, including the deadly form of melanomas (de Gruijl, 2002). Moreover, UV light (used in both photo-caged PROTACs and photo-switch PROTACs) and visible light (used in photo-switch PROTACs) have limited penetration ability, thus making those light-controllable PROTACs only suitable for several types of cancer that can be accessed easily by light, such skin cancer or leukemia. To overcome such disadvantages, further effects should be focused on adopting other light source rather than UV light to trigger the photo-cage or photo-switch process. To this end, visible light or near-infrared light has longer wavelength and less energy than UV to trigger potential DNA damage (Mouret et al., 2006; Cadet and Douki, 2011), making them more suitable to be the cage group on PROTACs. More importantly, several photo-cage group with red and near-infrared light sensitivity have been developed recently (Vorobev and Moskalensky, 2020), including N-NO (Nakagawa, 2016) and benzoquinone-based photocage (Chen and Steinmetz, 2006; Wang and Kalow, 2018; Alabugin, 2019). Furthermore, other endogenous cues in cancers such as those cancer-specific antigens or receptors should be also useful for targeting delivery of PROTAC to cancer cells, thus eliminating possible toxic issue to normal tissues/cells (Liu et al., 2019; Saw and Song, 2019).

Another potential disadvantage of light-controllable PROTACs is due to their permeability. Compared with small molecule drug which is usually less than 500 Da, according to the Lipinski’s rule of five (Lipinski et al., 2001), standard PROTACs are usually more than 600 Da and these light-controllable PROTACs are usually near 1,000 Da. The relatively large molecule weight might compromise the pharmacokinetic and pharmacodynamic parameters of light-controllable PROTACs. To date, in most *in vivo* study, PROTACs are administrated by Raina et al. (2016); Ohoka et al. (2017b), Sun B. et al. (2018), intraperitoneally (Winter et al., 2015; Zhou et al., 2018; Gao et al., 2020) or intravenously (Mares et al., 2020) injection. Thus, it still warrants further in-depth investigation on optimization the pharmaceutical properties of PROTAC to make it possible for orally administered.

Finally, in clinic, there is lack of clear boundary between tumor tissues and adjunct normal tissues, making it hard to only activate these light-controllable PROTACs at the tumor tissues/cells. An alternative approach for controllable action of PROTACs in cancer cells could be taking advantage of cancer-specific receptors or transporters, such as HER2 and FOLR1 (Scaranti et al., 2020) for the guided delivery of PROTACs into cancer, but not normal cells. To this end, other types of third generation PROTACs, including antibody-conjugated PROTACs (Dragovich et al., 2020, 2021a,b; Maneiro et al., 2020; Pillow et al., 2020) and folate-PROTAC (Liu et al., 2021), have been recently developed, which specifically deliver PROTAC to cancer cells, thus avoiding potential toxicity to normal cells. Compared with the light-controllable PROTACs, folate-PROTAC (Liu et al., 2021) have relatively higher molecule weight of over 1,000 Da, and antibody-conjugated PROTACs (Dragovich et al., 2020, 2021a,b; Maneiro et al., 2020; Pillow et al., 2020) are macromolecule drug that could only be administrated by injection. Taken together, further studies are needed to make these third generation PROTACs (light-controllable PROTACs, antibody-conjugated PROTACs and folate-PROTAC) more practical in clinic.

## Author Contributions

All authors listed have made a substantial, direct and intellectual contribution to the work, and approved it for publication.

## Conflict of Interest

WW is a co-founder and consultant for the ReKindle Therapeutics. The remaining authors declare that the research was conducted in the absence of any commercial or financial relationships that could be construed as a potential conflict of interest.
