# Supplementary material for: Light-Controllable PROTACs for Temporospatial Control of Protein Degradation
Source: Front Cell Dev Biol. 2021 Jul 19;9:678077. doi: 10.3389/fcell.2021.678077 (PMC8326567; doi:10.3389/fcell.2021.678077)
Supplement: Supplementary file 1 [file Table_1.docx]

**Supplemental Material**

**Light-controllable PROTACs for temporospatial control of protein degradation**

Jing Liu^1, *^, Yunhua Peng^1, *^, Wenyi Wei ^1, #^

^1^Department of Pathology, Beth Israel Deaconess Medical Center, Harvard Medical School, Boston, MA, USA

^*^ Jing Liu and Yunhua Peng contributed equally to this work.

#: To whom correspondence should be addressed:

Dr. Wenyi Wei, [wwei2@bidmc.harvard.edu](mailto:wwei2@bidmc.harvard.edu)

| **Supplemental Table 1. Representative PROTACs and their biological functions.** | | | |
| --- | --- | --- | --- |
| POI | PROTAC | Ligand, E3 and Cellular Functions | Ref. |
| ALK | TD-004 | - ceritinib; VHL. - induces the degradation of the NPM-ALK and the EML4-ALK fusion proteins; - inhibits the proliferation of SU-DHL-1 and H3122 cells; - reduces the tumor growth in H3122 xenograft model. | [[1](#_ENREF_1)] |
| ALK | Compd. 9 and 11 | - TAE684 or LDK378; pomalidomide; CRBN. - induces the degradation of NPM-ALK and EML4-ALK; - inhibits the proliferation of H3122, Karpas 229 and SU-DHL-1 cells. | [[2](#_ENREF_2)] |
| ALK | SIAIS117 | - Brigatinib; VHL-1; VHL. - induces the degradation of NPM-ALK, EML4-ALK and the ALK-G1202R mutant; - inhibits the proliferation of SR and H2228 cells, as well as 293T cells expressing ALK-G1202R-resistant proteins. | [[3](#_ENREF_3)] |
| AR | ARV-110 | - pomalidomide; CRBN. - completely degrades AR and various AR mutants; - inhibits AR-dependent cell proliferation and induces potent apoptosis in VCaP cells; - reduces AR protein abundance in xenograft VCaP tumor model. | [[4](#_ENREF_4)] |
| AR | ARCC-4 | - enzalutamide; VHL. - degrades wild type AR and clinically relevant mutant forms of AR; - inhibits the proliferation of PRCA cells; - retains anti-proliferative effect in a high androgen environment. | [[5](#_ENREF_5)] |
| AR | ARD-69 | - enzalutamide; VHL. - induces the degradation of AR; - inhibits the proliferation of AR+ PRCA cells (LNCaP, VCaP and 22Rv1); - reduces AR protein abundance in VCaP xenograft tumor tissue (IP injection). | [[6](#_ENREF_6)] |
| AKT1/2/3 | INY-03-041 | - GDC-0068; lenalidomide; CRBN. - induces the degradation of AKT1/2/3; - inhibits the proliferation of MOLT4, LNCaP and BRCA cells (ZR-75-1, T47D, MCF-7, MDA-MB-468 and HCC1937). | [[7](#_ENREF_7)] |
| AURORA-A | JB170 | - alisertib, thalidomide; CRBN. - induces rapid, durable and highly specific degradation of AURORA-A; - causes an S-phase defect, which is not observed upon kinase inhibition; - induces rampant apoptosis in cancer cell lines. | [[8](#_ENREF_8)] |
| BCL2/MCL-1 | C3, C5 | - S1-6 or Nap-1; Pomalidomide; CRBN. - induces the degradation of Mcl-1 and Bcl-2; - induces lethality in H23 cells. | [[9](#_ENREF_9)] |
| BCL6 | Compd. 15 | - pomalidomide; CRBN. - induces the degradation of BCL6; - weak anti-proliferative response in DLBCL OCI-Ly1 cells. | [[10](#_ENREF_10)] |
| BCL-XL | XZ739 | - ABT-263; pomalidomide; CRBN. - induce the degradation of BCL-XL; - inhibits the proliferation MOLT-4 cells more potent than ABT-263; - less toxic to human platelets than ABT-263; - induces apoptosis of MOLT-4 cells. | [[11](#_ENREF_11)] |
| BCL-XL | PZ15227 (PZ) | - ABT-263; pomalidomide; CRBN. - induces the degradation of BCL-XL; - less toxic to platelets, but equally or slightly more potent against senescent cells; - effectively clears senescent cells and rejuvenates tissue stem and progenitor cells in naturally aged mice without causing severe thrombocytopenia. | [[12](#_ENREF_12)] |
| BCR-ABL | SIAIS178 | - dasatinib; VHL 1; VHL. - induces the degradation of BCR-ABL and several clinically relevant resistance-conferring mutant forms of BCR-ABL; - achieves significant growth inhibition of BCR-ABL+ leukemic cells *in vitro;* - induces substantial tumor regression against K562 xenograft tumors *in vivo.* | [[13](#_ENREF_13)] |
| BCR-ABL | GMB-475 | - VHL. - induces rapid degradation of BCR-ABL and inhibition of downstream biomarkers, such as STAT5 in CML K562 cells and murine Ba/F3 cells expressing BCR-ABL1; - inhibits the proliferation of certain clinically relevant BCR-ABL1 kinase domain point mutants and further sensitizes Ba/F3 BCR-ABL1 cells to inhibition by imatinib, while demonstrating no toxicity toward Ba/F3 parental cells; - reduces the viability and increases apoptosis of primary CML CD34+ cells, with no effect on healthy CD34+ cells; - degrades BCR-ABL1 and reduces cell viability in primary CML stem cells. | [[14](#_ENREF_14)] |
| BCR-ABL | DAS-6-2-2-6-CRBN | - dasatinib or bosutinib; pomalidomide; CRBN. - induces the degradation of ABL and ABL/BCR; - inhibits the proliferation of ABL/BCR-expressing K562 cells. | [[15](#_ENREF_15)] |
| BRAF | Compd. 2 | - RGS; pomalidomide; CRBN. - induces the degradation of BRAF; - induces apoptosis of MCF-7 cells. | [[16](#_ENREF_16)] |
| BRAF | Compd. 12 and 23 | - vemurafenib or BI882370; thalidomide; CRBN. - induces selective degradation of BRAF-V600E, but not wild-type BRAF; - inhibits the proliferation of A375 and HT-29 cells. | [[17](#_ENREF_17)] |
| BRD4 | dBET1 | - JQ1; pomalidomide; CRBN. - induces the degradation of BRD4; - inhibits the proliferation of MV4-11 cells. | [[18](#_ENREF_18)] |
| BRD4 | ARV-825 | - JQ1; pomalidomide; CRBN. - induces the degradation of BRD4 in Burkitt's lymphoma (BL) cells; - inhibits the proliferation and induces apoptosis in BL cells. | [[19](#_ENREF_19)] |
| BRD4 | dBET6 | - JQ1; pomalidomide; CRBN. - induces the degradation of BRD4; - inhibits the proliferation of T-ALL cells; - prompts a collapse of global elongation that phenocopies CDK9 inhibition. | [[20](#_ENREF_20)] |
| BRD4 | MZ1 | - JQ1, VHL. - induces reversible, long-lasting and unexpectedly selective removal of BRD4 over BRD2 and BRD3. | [[21](#_ENREF_21)] |
| BRD7/9 | VZ185 | - VHL. - degrades BRD7 and BRD9; - inhibits the proliferation of EOL-1 and A-204 cancer cells. | [[22](#_ENREF_22)] |
| BTK | Compd. 10 | - pomalidomide; CRBN. - induces the degradation of BTK in Ramos cells and in Rat’s spleen. | [[23](#_ENREF_23)] |
| BTK and BTK-C481S | P13I | - ibrutinib; pomalidomide; CRBN. - induces BTK degradation in human ABC-DLBCL, HBL-1 cells and other NHL cell lines including MCL (Mino cells) and MM cell lines; - inhibits the proliferation of HBL-1 cells expressing BTK-C481S. | [[24](#_ENREF_24)] |
| BTK-C481S | L18I | - ibrutinib; lenalidomide; CRBN. - induces the degradation of BTK-C481S and other C481 mutates; - inhibits the growth of DLBCL and MCL cells; - induces rapid tumor regression of C481S BTK HBL-1 xenograft tumors. | [[25](#_ENREF_25)] |
| BTK | CJH-005-067 and DD-04-015 | - bosutinib or RN486; pomalidomide; CRBN. - induces the degradation of BTK; - inhibits the proliferation of TMD8 DLBCL cell. | [[26](#_ENREF_26)] |
| CDC20 | CP5V | - Apcin; VHL. - degrades Cdc20 in MCF7 and MDA-MB-231 BRCA cells; - leads to significant inhibition of BRCA cell proliferation and re-sensitization of Taxol-resistant cell lines; - suppresses breast tumor progression in 4T1 xenograft mouse model. | [[27](#_ENREF_27)] |
| CDK2 | CPS1/2/3 | - JNJ-7706621; pomalidomide; CRBN. - promotes rapid and potent degradation of CDK2, but not other CDKs; - induces remarkable differentiation of AML cell lines and primary patient cells. | [[28](#_ENREF_28)] |
| CDK2 and CDK5 | TMX-2172 | - TMX-2039; pomalidomide; CRBN. - induces the degradation of CDK2 and CDK5; - inhibits the proliferation of OVCAR8 cells. | [[29](#_ENREF_29)] |
| CDK2 and CDK9 | Compd. F3 | - FN-1501; pomalidomide; CRBN. - induces the degradation of CDK2 and CDK9; - inhibits the proliferation of PC-3 cells. | [[30](#_ENREF_30)] |
| CDK4 | BSJ-03-132 | - abemaciclib; pomalidomide; CRBN. - induces the degradation of CDK4. | [[31](#_ENREF_31)] |
| CDK4 and CDK6 | BSJ-02-162; BSJ-03-204 | - palbociclib; pomalidomide ; CRBN. - induces the degradation of CDK4 and CDK6; - inhibits the proliferation of MCL cell lines. | [[31](#_ENREF_31)] |
| CDK4 and CDK6 | pal-pom | - palbociclib; pomalidomide ; CRBN. - degrades CDK4 and CDK6. | [[32](#_ENREF_32)] |
| CDK6 | Degrader 6 | - palbociclib; pomalidomide ; CRBN. - induces the degradation of CDK6, but not CDK4 or other CDKs. | [[33](#_ENREF_33)] |
| CDK6 | YX-2-107 | - palbociclib; thalidomide; CRBN. - promotes the degradation of CDK6 over CDK4 in Ph+ ALL cells; - suppresses S-phase cells; - suppresses leukemia burden in mice injected with primary Ph+ ALL cells. | [[34](#_ENREF_34)] |
| CDK6 | BSJ-03-123 | - palbociclib; pomalidomide; CRBN. - induces the degradation of CDK6; - inhibits the proliferation of AML cell lines. | [[35](#_ENREF_35)] |
| CDK6 | CP-10 | - palbociclib; pomalidomide; CRBN. - inhibits the proliferation of cancer cells (MM.1S, Mino, HL-60, JeKo-1); - induces the degradation of CDK6-WT, D163G and S178P mutants. | [[36](#_ENREF_36)] |
| CDK9 | Compd. 3 | - aminopyrazole; thalidomide; CRBN. - induces the degradation of CDK9 in HCT116 cells. | [[37](#_ENREF_37)] |
| CDK9 | 11c | - flavopiridol; pomalidomide; CRBN. - selectively degrades CDK9; - inhibits the proliferation of CDK9-overexpressed cancer cells. | [[38](#_ENREF_38)] |
| EGFR | PROTAC 2 | - lenalidomide; CRBN. - induces degradation of EGFR in HCC827 cells; - induces the apoptosis of HCC827 cells and arrest the cells in G1 phase. | [[39](#_ENREF_39)] |
| EGFR | PROTAC 10 | - VHL ligand; VHL. - induces the degradation of EGFR in HCC827 cells; - induces the apoptosis of HCC827 cells and arrest the cells in G1 phase. | [[40](#_ENREF_40)] |
| EGFR | MS39 | - gefitinib; VHL ligand; VHL. - induces the degradation of mutant, but not wild-type EGFR; - suppresses the growth of lung cancer cells (HCC-827 with EGFR-e19d and H3255 cells with EGFR-L858R mutant). | [[41](#_ENREF_41)] |
| EGFR mutant | 14o | - XTF262; VHL. - effectively and selectively degraded EGFRL858R/T790M, but not wild-type EGFR. | [[42](#_ENREF_42)] |
| EGFR mutant | MS154 | - gefitinib; pomalidomide; CRBN. - induces the degradation of mutant, but not wild-type EGFR; - suppresses the growth of lung cancer cells (HCC-827 with EGFR-e19d and H3255 cells with EGFR-L858R mutant). | [[41](#_ENREF_41)] |
| EGFR mutant | DDC-01-163 | - pomalidomide; CRBN. - allosteric EGFR degrader; - selective degrades clinically relevant EGFR mutants; - selectively inhibits the proliferation of L858R/T790M (L/T) mutant Ba/F3 cells but not wildtype EGFR Ba/F3 cells; - inhibits the proliferation of osimertinib-resistant cells with L/T/C797S and L/T/L718Q EGFR mutations. | [[39](#_ENREF_39)] |
| ER | ERD-308 | - tamoxifen; VHL. - degrades ER in MCF-7 and T47D ER+ BRCA cell lines; - more effective in inhibition of cell proliferation than fulvestrant in MCF-7 cells. | [[43](#_ENREF_43)] |
| ER | ARV-471 | - thalidomide; CRBN. - robustly degrades ER in ER-positive breast cancer cell lines; - decreases the expression of classically-regulated ER-target genes (PR, GREB1, TFF); - inhibits the proliferation of ER-dependent cell lines (MCF7, T47D); - degrades clinically-relevant ER variants (Y537S and D538G) and inhibits growth of cell lines expressing those variants; - degrades rat uterine ER in an immature rat uterotrophic model; - daily, oral-administration leads to tumor volume regression of estradiol-dependent MCF7 xenografts and tumor ER protein reduction; - inhibits the growth and reduces mutant ER protein levels in an ER-Y537S, hormone-independent patient-derived xenograft model. | [[44](#_ENREF_44)] |
| ERR | Compd. 6c | - XCT790; VHL. - capable of specifically degrading ERRα. | [[45](#_ENREF_45)] |
| FAK | FC-11 | - PF562271; thalidomide; CRBN. - induces a potent and reversible FAK degradation in reproductive tissues of male mice. | [[46](#_ENREF_46)] |
| FAK | PROTAC-3 | - defactinib; VHL. - degrades FAK; - inhibits FAK signaling; - represses FAK-mediated cell migration and invasion in MDA-MB-231 cells. | [[47](#_ENREF_47)] |
| FLT3 | TL13-117; TL13-149 | - AC220; pomalidomide; CRBN. - induces the degradation of FLT3; - inhibits the proliferation of MOLM-14 and MV4-11 cells. | [[26](#_ENREF_26)] |
| HDAC1/2/3 | Compd. 4 | - benzamide; VHL. - degrades HDAC 1/2/3; - increases histone acetylation levels; - compromises the viability of colon cancer HCT116 cells. | [[48](#_ENREF_48)] |
| HDAC6 | dHDAC6-9c | - a pan-HDAC inhibitor; pomalidomide; CRBN. - induces the degradation of HDAC6 in MCF7 and MM.1S cells. | [[49](#_ENREF_49)] |
| HDAC6 | dHDAC6-12d | - nexturastat A; pomalidomide; CRBN. - induces the degradation of HDAC6; - inhibits the proliferation of multiple myeloma cells. | [[50](#_ENREF_50)] |
| HDAC6 | NP8 | - nexturastat A; pomalidomide; CRBN. - induces the degradation of HDAC6; - inhibits the proliferation of multiple myeloma cells. | [[51](#_ENREF_51)] |
| HDAC6 | Compd. 3j | - nexturastat A; VHL. - degrades HDAC6 in human MM1S cells and mouse 4935 cells. | [[52](#_ENREF_52)] |
| HMGCR | P22A | - atorvastatin; pomalidomide; CRBN. - reduces HMGCR protein in SRD15 cells; - blocks cholesterol biosynthesis potently with less compensatory upregulation of HMGCR. | [[53](#_ENREF_53)] |
| KRAS | LC-2 | - MRTX849; VHL. - covalently binds and degrades KRAS-G12C; - leads to suppression of the MAPK signaling in both homozygous and heterozygous KRAS-G12C cell lines. | [[54](#_ENREF_54)] |
| IRAK4 | Compd. 9 | - PF06650833; VHL. - induces the degradation of IRAK4; - induces the inhibition of multiple cytokines in PBMCs, but not in IL-1β stimulated human dermal fibroblasts. | [[55](#_ENREF_55)] |
| MDM2 | PROTAC 8 | - MI-1061; lenalidomide; CRBN. - reduces MDM2 protein level; - exhibits enhanced efficacy in the RS4;11 xenograft model relative to MI-1061. | [[56](#_ENREF_56)] |
| MDM2 | MD-224 | - MI-1061; lenalidomide; CRBN. - induces rapid degradation of MDM2 at concentrations <1 nM in human leukemia cells. - inhibits the growth of RS4;11 cells and leukemia cell lines; - achieves complete and durable tumor regression *in vivo* in the RS4;11 xenograft tumor model in mice. | [[57](#_ENREF_57)] |
| MEK1/2 | Compd. 3 | - refametinib virtual analog 1; VHL ligand; VHL. - reduces MEK1 and MEK2 protein levels; - inhibits the proliferation of A375 cells. | [[58](#_ENREF_58)] |
| MEK1/2 | MS432 | - PD0325901; VHL. - induces the degradation of MEK1/2; - inhibits the proliferation of HT-29 colorectal cancer and SK-MEL-28 melanoma cell, phenocopied by MEK1/2 knockdown. | [[59](#_ENREF_59)] |
| PARP1 | iRucaparib | - rucaparib; pomalidomide; CRBN. - selectively targets PARP1 for degradation; - inhibits PARylation-mediated signaling events downstream of PARP1; - protects cells from genotoxicity-induced cell death. | [[60](#_ENREF_60)] |
| PARP1 | compound 3 | - VHL. - induces significant PARP1 cleavage; - induces programmed cell death in MDA-MB-231 cells. | [[61](#_ENREF_61)] |
| PCAF/GCN5 | GSK983 | - GSK4027; pomalidomide; CRBN. - degrades PCAF/GCN5; - potently modulates the expression of multiple inflammatory mediators in LPS-stimulated macrophages and dendritic cells. | [[62](#_ENREF_62)] |
| PRC2 (EED/EZH2/SUZ12) | UNC6852 | - EED226; VHL. - induces the degradation of PRC2 components, EED, EZH2, and SUZ12; - blocks the histone methyltransferase activity of EZH2, decreasing H3K27me3 levels in HeLa cells and diffuse large B cell lymphoma (DLBCL) cells containing EZH2 gain-of-function mutations; - degrades both wild-type and mutant EZH2; - displays anti-proliferative effects. | [[63](#_ENREF_63)] |
| P38 | SJFα and SJFδ | - foretinib; VHL. - induces the degradation of p38α or p38δ in MDA-MB-231 cells. | [[64](#_ENREF_64)] |
| PI3K | Compounds D-F | - ZSTK474; pomalidomide; CRBN. - induces remarkable PI3K degradation and down-regulation of downstream signaling (p-Akt, p-S6K and p-GSK-3β) in HepG2 cells; - inhibits the proliferation of HepG2 cells by induction of autophagy instead of apoptosis or cell cycle arrest. | [[65](#_ENREF_65)] |
| RIPK2 | PROTAC 6 | - aminobenzothiazole-quinoline; VHL ligand; VHL. - induces potent degradation of RIPK2 in human PBMCs; - inhibits cytokine release in human disease tissue; - *in vivo* degradation of endogenous RIPK2 in rats at low doses with persisted PD. | [[66](#_ENREF_66)] |
| RTK | PROTAC 1 and 7 | - lapatinib and foretinib; VHL. - PROTAC 1 degrades HER2 in SKBR3 and OVCAR8 cells; - PROTAC 7 degrades c-MET in MDA-MB-231 cells. | [[67](#_ENREF_67)] |
| SGK3 | SGK3-PROTAC1 | - 308-R; VH032; VHL. - induces the degradation of SGK3 but not SGK1 and SGK2; - suppresses the proliferation of ZR-75-1 and CAMA-1 BRCA cells treated with a PI3K inhibitor (GDC0941); - restores the sensitivity of SGK3-dependent ZR-75-1 and CAMA-1 BRCA cells to Akt (AZD5363) and PI3K (GDC0941) inhibitors. | [[68](#_ENREF_68)] |
| SHP2 | SHP2-D26 | - SHP099; VHL. - degrades SHP2 in esophageal cancer KYSE520 cells and MV4-11 AML cells; - more potent in inhibition of p-ERK and of cell growth. | [[69](#_ENREF_69)] |
| SIRT2 | Compd. 12 | - SirReals; thalidomide; CRBN. - induces isotype-selective Sirt2 degradation; - causes hyperacetylation of the microtubule network coupled with enhanced process elongation in HeLa cells. | [[70](#_ENREF_70)] |
| SMAD 3 | SMAD PROTAC | - induces the degradation of Smad3 in ACHN cells; - inhibits the upregulation of fibronectin and Collagen I induced by TGF-β1 in both renal fibroblast and mesangial cells. | [[71](#_ENREF_71)] |
| SMARCA2/4 and PBRM1 | ACBI1 | - 2-(6-aminopyridazin-3-yl)phenols; VHL. - inhibits the proliferation and induces cell death in SMARCA4 mutant cancer cells and in AML cells. | [[72](#_ENREF_72)] |
| STAT3 | SD-36 | - SI-109; thalidomide; CRBN. - induces rapid degradation of STAT3, but not other STAT proteins; - inhibits cell growth in leukemia and lymphoma cell lines with high levels of p-STAT3; - a single dose results in complete STAT3 protein degradation in xenograft tumor tissue and normal mouse tissues. | [[73](#_ENREF_73), [74](#_ENREF_74)] |
| TRIM24 | dTRIM24 | - IACS-9571; VHL. - elicits potent and selective degradation of TRIM24; - induces anti-proliferative response. | [[75](#_ENREF_75)] |
| WEE1 | ZNL-02-096 | - AZD1775; pomalidomide; CRBN. - degrades Wee1 while sparing PLK1; - induces G2/M accumulation; - synergizes with Olaparib in ovarian cancer cells (OVCAR8, COV283, and Kuramochi). | [[76](#_ENREF_76)] |

**References:**

1. Kang, C.H., D.H. Lee, C.O. Lee, J. Du Ha, C.H. Park, and J.Y. Hwang (2018). Induced protein degradation of anaplastic lymphoma kinase (ALK) by proteolysis targeting chimera (PROTAC)*.* *Biochem Biophys Res Commun* 505, 542-547. doi: 10.1016/j.bbrc.2018.09.169.

2. Powell, C.E., Y. Gao, L. Tan, K.A. Donovan, R.P. Nowak, A. Loehr, et al. (2018). Chemically Induced Degradation of Anaplastic Lymphoma Kinase (ALK)*.* *J Med Chem* 61, 4249-4255. doi: 10.1021/acs.jmedchem.7b01655.

3. Sun, N., C. Ren, Y. Kong, H. Zhong, J. Chen, Y. Li, et al. (2020). Development of a Brigatinib degrader (SIAIS117) as a potential treatment for ALK positive cancer resistance*.* *Eur J Med Chem* 193, 112190. doi: 10.1016/j.ejmech.2020.112190.

4. Taavi Neklesa, Lawrence B. Snyder, Ryan R. Willard, Nicholas Vitale, Kanak Raina, Jennifer Pizzano, et al. (2018). Abstract 5236: ARV-110: An androgen receptor PROTAC degrader for prostate cancer*.* *Cancer Res*. doi: 10.1158/1538-7445.

5. Salami, J., S. Alabi, R.R. Willard, N.J. Vitale, J. Wang, H. Dong, et al. (2018). Androgen receptor degradation by the proteolysis-targeting chimera ARCC-4 outperforms enzalutamide in cellular models of prostate cancer drug resistance*.* *Commun Biol* 1, 100. doi: 10.1038/s42003-018-0105-8.

6. Han, X., C. Wang, C. Qin, W. Xiang, E. Fernandez-Salas, C.Y. Yang, et al. (2019). Discovery of ARD-69 as a Highly Potent Proteolysis Targeting Chimera (PROTAC) Degrader of Androgen Receptor (AR) for the Treatment of Prostate Cancer*.* *J Med Chem* 62, 941-964. doi: 10.1021/acs.jmedchem.8b01631.

7. You, I., E.C. Erickson, K.A. Donovan, N.A. Eleuteri, E.S. Fischer, N.S. Gray, et al. (2020). Discovery of an AKT Degrader with Prolonged Inhibition of Downstream Signaling*.* *Cell chemical biology* 27, 66-73 e7. doi: 10.1016/j.chembiol.2019.11.014.

8. Adhikari, B., J. Bozilovic, M. Diebold, J.D. Schwarz, J. Hofstetter, M. Schroder, et al. (2020). PROTAC-mediated degradation reveals a non-catalytic function of AURORA-A kinase*.* *Nat Chem Biol* 16, 1179-1188. doi: 10.1038/s41589-020-00652-y.

9. Wang, Z., N. He, Z. Guo, C. Niu, T. Song, Y. Guo, et al. (2019). Proteolysis Targeting Chimeras for the Selective Degradation of Mcl-1/Bcl-2 Derived from Nonselective Target Binding Ligands*.* *J Med Chem* 62, 8152-8163. doi: 10.1021/acs.jmedchem.9b00919.

10. McCoull, W., T. Cheung, E. Anderson, P. Barton, J. Burgess, K. Byth, et al. (2018). Development of a Novel B-Cell Lymphoma 6 (BCL6) PROTAC To Provide Insight into Small Molecule Targeting of BCL6*.* *ACS Chem Biol* 13, 3131-3141. doi: 10.1021/acschembio.8b00698.

11. Zhang, X., D. Thummuri, X. Liu, W. Hu, P. Zhang, S. Khan, et al. (2020). Discovery of PROTAC BCL-XL degraders as potent anticancer agents with low on-target platelet toxicity*.* *Eur J Med Chem* 192, 112186. doi: 10.1016/j.ejmech.2020.112186.

12. He, Y., X. Zhang, J. Chang, H.N. Kim, P. Zhang, Y. Wang, et al. (2020). Using proteolysis-targeting chimera technology to reduce navitoclax platelet toxicity and improve its senolytic activity*.* *Nat Commun* 11, 1996. doi: 10.1038/s41467-020-15838-0.

13. Zhao, Q., C. Ren, L. Liu, J. Chen, Y. Shao, N. Sun, et al. (2019). Discovery of SIAIS178 as an Effective BCR-ABL Degrader by Recruiting Von Hippel-Lindau (VHL) E3 Ubiquitin Ligase*.* *J Med Chem* 62, 9281-9298. doi: 10.1021/acs.jmedchem.9b01264.

14. Burslem, G.M., A.R. Schultz, D.P. Bondeson, C.A. Eide, S.L. Savage Stevens, B.J. Druker, et al. (2019). Targeting BCR-ABL1 in Chronic Myeloid Leukemia by PROTAC-Mediated Targeted Protein Degradation*.* *Cancer Res* 79, 4744-4753. doi: 10.1158/0008-5472.CAN-19-1236.

15. Lai, A.C., M. Toure, D. Hellerschmied, J. Salami, S. Jaime-Figueroa, E. Ko, et al. (2016). Modular PROTAC Design for the Degradation of Oncogenic BCR-ABL*.* *Angew Chem Int Ed Engl* 55, 807-10. doi: 10.1002/anie.201507634.

16. Chen, H., F. Chen, S. Pei, and S. Gou (2019). Pomalidomide hybrids act as proteolysis targeting chimeras: Synthesis, anticancer activity and B-Raf degradation*.* *Bioorg Chem* 87, 191-199. doi: 10.1016/j.bioorg.2019.03.035.

17. Han, X.R., L. Chen, Y. Wei, W. Yu, Y. Chen, C. Zhang, et al. (2020). Discovery of Selective Small Molecule Degraders of BRAF-V600E*.* *J Med Chem* 63, 4069-4080. doi: 10.1021/acs.jmedchem.9b02083.

18. Winter, G.E., D.L. Buckley, J. Paulk, J.M. Roberts, A. Souza, S. Dhe-Paganon, et al. (2015). DRUG DEVELOPMENT. Phthalimide conjugation as a strategy for in vivo target protein degradation*.* *Science* 348, 1376-81. doi: 10.1126/science.aab1433.

19. Lu, J., Y. Qian, M. Altieri, H. Dong, J. Wang, K. Raina, et al. (2015). Hijacking the E3 Ubiquitin Ligase Cereblon to Efficiently Target BRD4*.* *Chem Biol* 22, 755-63. doi: 10.1016/j.chembiol.2015.05.009.

20. Winter, G.E., A. Mayer, D.L. Buckley, M.A. Erb, J.E. Roderick, S. Vittori, et al. (2017). BET Bromodomain Proteins Function as Master Transcription Elongation Factors Independent of CDK9 Recruitment*.* *Mol Cell* 67, 5-18 e19. doi: 10.1016/j.molcel.2017.06.004.

21. Zengerle, M., K.H. Chan, and A. Ciulli (2015). Selective Small Molecule Induced Degradation of the BET Bromodomain Protein BRD4*.* *ACS Chem Biol* 10, 1770-7. doi: 10.1021/acschembio.5b00216.

22. Zoppi, V., S.J. Hughes, C. Maniaci, A. Testa, T. Gmaschitz, C. Wieshofer, et al. (2019). Iterative Design and Optimization of Initially Inactive Proteolysis Targeting Chimeras (PROTACs) Identify VZ185 as a Potent, Fast, and Selective von Hippel-Lindau (VHL) Based Dual Degrader Probe of BRD9 and BRD7*.* *J Med Chem* 62, 699-726. doi: 10.1021/acs.jmedchem.8b01413.

23. Zorba, A., C. Nguyen, Y. Xu, J. Starr, K. Borzilleri, J. Smith, et al. (2018). Delineating the role of cooperativity in the design of potent PROTACs for BTK*.* *Proc Natl Acad Sci U S A* 115, E7285-E7292. doi: 10.1073/pnas.1803662115.

24. Sun, Y., X. Zhao, N. Ding, H. Gao, Y. Wu, Y. Yang, et al. (2018). PROTAC-induced BTK degradation as a novel therapy for mutated BTK C481S induced ibrutinib-resistant B-cell malignancies*.* *Cell research* 28, 779-781. doi: 10.1038/s41422-018-0055-1.

25. Sun, Y., N. Ding, Y. Song, Z. Yang, W. Liu, J. Zhu, et al. (2019). Degradation of Bruton's tyrosine kinase mutants by PROTACs for potential treatment of ibrutinib-resistant non-Hodgkin lymphomas*.* *Leukemia* 33, 2105-2110. doi: 10.1038/s41375-019-0440-x.

26. Huang, H.T., D. Dobrovolsky, J. Paulk, G. Yang, E.L. Weisberg, Z.M. Doctor, et al. (2018). A Chemoproteomic Approach to Query the Degradable Kinome Using a Multi-kinase Degrader*.* *Cell chemical biology* 25, 88-99 e6. doi: 10.1016/j.chembiol.2017.10.005.

27. Chi, J.J., H. Li, Z. Zhou, J. Izquierdo-Ferrer, Y. Xue, C.M. Wavelet, et al. (2019). A novel strategy to block mitotic progression for targeted therapy*.* *EBioMedicine* 49, 40-54. doi: 10.1016/j.ebiom.2019.10.013.

28. Wang, L., X. Shao, T. Zhong, Y. Wu, A. Xu, X. Sun, et al. (2021). Discovery of a first-in-class CDK2 selective degrader for AML differentiation therapy*.* *Nat Chem Biol* 17, 567-575. doi: 10.1038/s41589-021-00742-5.

29. Teng, M., J. Jiang, Z. He, N.P. Kwiatkowski, K.A. Donovan, C.E. Mills, et al. (2020). Development of CDK2 and CDK5 Dual Degrader TMX-2172*.* *Angew Chem Int Ed Engl* 59, 13865-13870. doi: 10.1002/anie.202004087.

30. Zhou, F., L. Chen, C. Cao, J. Yu, X. Luo, P. Zhou, et al. (2020). Development of selective mono or dual PROTAC degrader probe of CDK isoforms*.* *Eur J Med Chem* 187, 111952. doi: 10.1016/j.ejmech.2019.111952.

31. Jiang, B., E.S. Wang, K.A. Donovan, Y. Liang, E.S. Fischer, T. Zhang, et al. (2019). Development of Dual and Selective Degraders of Cyclin-Dependent Kinases 4 and 6*.* *Angew Chem Int Ed Engl* 58, 6321-6326. doi: 10.1002/anie.201901336.

32. Zhao, B. and K. Burgess (2019). PROTACs suppression of CDK4/6, crucial kinases for cell cycle regulation in cancer*.* *Chem Commun (Camb)* 55, 2704-2707. doi: 10.1039/c9cc00163h.

33. Rana, S., M. Bendjennat, S. Kour, H.M. King, S. Kizhake, M. Zahid, et al. (2019). Selective degradation of CDK6 by a palbociclib based PROTAC*.* *Bioorg Med Chem Lett* 29, 1375-1379. doi: 10.1016/j.bmcl.2019.03.035.

34. De Dominici, M., P. Porazzi, Y. Xiao, A. Chao, H.Y. Tang, G. Kumar, et al. (2020). Selective inhibition of Ph-positive ALL cell growth through kinase-dependent and -independent effects by CDK6-specific PROTACs*.* *Blood* 135, 1560-1573. doi: 10.1182/blood.2019003604.

35. Brand, M., B. Jiang, S. Bauer, K.A. Donovan, Y. Liang, E.S. Wang, et al. (2019). Homolog-Selective Degradation as a Strategy to Probe the Function of CDK6 in AML*.* *Cell chemical biology* 26, 300-306 e9. doi: 10.1016/j.chembiol.2018.11.006.

36. Su, S., Z. Yang, H. Gao, H. Yang, S. Zhu, Z. An, et al. (2019). Potent and Preferential Degradation of CDK6 via Proteolysis Targeting Chimera Degraders*.* *J Med Chem* 62, 7575-7582. doi: 10.1021/acs.jmedchem.9b00871.

37. Robb, C.M., J.I. Contreras, S. Kour, M.A. Taylor, M. Abid, Y.A. Sonawane, et al. (2017). Chemically induced degradation of CDK9 by a proteolysis targeting chimera (PROTAC)*.* *Chem Commun (Camb)* 53, 7577-7580. doi: 10.1039/c7cc03879h.

38. Bian, J., J. Ren, Y. Li, J. Wang, X. Xu, Y. Feng, et al. (2018). Discovery of Wogonin-based PROTACs against CDK9 and capable of achieving antitumor activity*.* *Bioorg Chem* 81, 373-381. doi: 10.1016/j.bioorg.2018.08.028.

39. Jang, J., C. To, D.J.H. De Clercq, E. Park, C.M. Ponthier, B.H. Shin, et al. (2020). Mutant-Selective Allosteric EGFR Degraders are Effective Against a Broad Range of Drug-Resistant Mutations*.* *Angew Chem Int Ed Engl* 59, 14481-14489. doi: 10.1002/anie.202003500.

40. Zhang, H., H.Y. Zhao, X.X. Xi, Y.J. Liu, M. Xin, S. Mao, et al. (2020). Discovery of potent epidermal growth factor receptor (EGFR) degraders by proteolysis targeting chimera (PROTAC)*.* *Eur J Med Chem* 189, 112061. doi: 10.1016/j.ejmech.2020.112061.

41. Cheng, M., X. Yu, K. Lu, L. Xie, L. Wang, F. Meng, et al. (2020). Discovery of Potent and Selective Epidermal Growth Factor Receptor (EGFR) Bifunctional Small-Molecule Degraders*.* *J Med Chem* 63, 1216-1232. doi: 10.1021/acs.jmedchem.9b01566.

42. Zhang, X., F. Xu, L. Tong, T. Zhang, H. Xie, X. Lu, et al. (2020). Design and synthesis of selective degraders of EGFR(L858R/T790M) mutant*.* *Eur J Med Chem* 192, 112199. doi: 10.1016/j.ejmech.2020.112199.

43. Hu, J., B. Hu, M. Wang, F. Xu, B. Miao, C.Y. Yang, et al. (2019). Discovery of ERD-308 as a Highly Potent Proteolysis Targeting Chimera (PROTAC) Degrader of Estrogen Receptor (ER)*.* *J Med Chem* 62, 1420-1442. doi: 10.1021/acs.jmedchem.8b01572.

44. JJ Flanagan, Y Qian, SM Gough, M Andreoli, M Bookbinder, G Cadelina, et al. (2019). Abstract P5-04-18: ARV-471, an oral estrogen receptor PROTAC degrader for breast cancer*.* *Cancer Res*. doi: 10.1158/1538-7445.

45. Peng, L., Z. Zhang, C. Lei, S. Li, X. Ren, Y. Chang, et al. (2019). Identification of New Small-Molecule Inducers of Estrogen-related Receptor alpha (ERRalpha) Degradation*.* *ACS Med Chem Lett* 10, 767-772. doi: 10.1021/acsmedchemlett.9b00025.

46. Gao, H., C. Zheng, J. Du, Y. Wu, Y. Sun, C. Han, et al. (2020). FAK-targeting PROTAC as a chemical tool for the investigation of non-enzymatic FAK function in mice*.* *Protein Cell* 11, 534-539. doi: 10.1007/s13238-020-00732-8.

47. Cromm, P.M., K.T.G. Samarasinghe, J. Hines, and C.M. Crews (2018). Addressing Kinase-Independent Functions of Fak via PROTAC-Mediated Degradation*.* *J Am Chem Soc* 140, 17019-17026. doi: 10.1021/jacs.8b08008.

48. Smalley, J.P., G.E. Adams, C.J. Millard, Y. Song, J.K.S. Norris, J.W.R. Schwabe, et al. (2020). PROTAC-mediated degradation of class I histone deacetylase enzymes in corepressor complexes*.* *Chem Commun (Camb)* 56, 4476-4479. doi: 10.1039/d0cc01485k.

49. Yang, K., Y. Song, H. Xie, H. Wu, Y.T. Wu, E.D. Leisten, et al. (2018). Development of the first small molecule histone deacetylase 6 (HDAC6) degraders*.* *Bioorg Med Chem Lett* 28, 2493-2497. doi: 10.1016/j.bmcl.2018.05.057.

50. Wu, H., K. Yang, Z. Zhang, E.D. Leisten, Z. Li, H. Xie, et al. (2019). Development of Multifunctional Histone Deacetylase 6 Degraders with Potent Antimyeloma Activity*.* *J Med Chem* 62, 7042-7057. doi: 10.1021/acs.jmedchem.9b00516.

51. An, Z., W. Lv, S. Su, W. Wu, and Y. Rao (2019). Developing potent PROTACs tools for selective degradation of HDAC6 protein*.* *Protein Cell* 10, 606-609. doi: 10.1007/s13238-018-0602-z.

52. Yang, K., H. Wu, Z. Zhang, E.D. Leisten, X. Nie, B. Liu, et al. (2020). Development of Selective Histone Deacetylase 6 (HDAC6) Degraders Recruiting Von Hippel-Lindau (VHL) E3 Ubiquitin Ligase*.* *ACS Med Chem Lett* 11, 575-581. doi: 10.1021/acsmedchemlett.0c00046.

53. Li, M.X., Y. Yang, Q. Zhao, Y. Wu, L. Song, H. Yang, et al. (2020). Degradation versus Inhibition: Development of Proteolysis-Targeting Chimeras for Overcoming Statin-Induced Compensatory Upregulation of 3-Hydroxy-3-methylglutaryl Coenzyme A Reductase*.* *J Med Chem* 63, 4908-4928. doi: 10.1021/acs.jmedchem.0c00339.

54. Bond, M.J., L. Chu, D.A. Nalawansha, K. Li, and C.M. Crews (2020). Targeted Degradation of Oncogenic KRAS(G12C) by VHL-Recruiting PROTACs*.* *ACS Cent Sci* 6, 1367-1375. doi: 10.1021/acscentsci.0c00411.

55. Nunes, J., G.A. McGonagle, J. Eden, G. Kiritharan, M. Touzet, X. Lewell, et al. (2019). Targeting IRAK4 for Degradation with PROTACs*.* *ACS Med Chem Lett* 10, 1081-1085. doi: 10.1021/acsmedchemlett.9b00219.

56. Wurz, R.P. and V.J. Cee (2019). Targeted Degradation of MDM2 as a New Approach to Improve the Efficacy of MDM2-p53 Inhibitors*.* *J Med Chem* 62, 445-447. doi: 10.1021/acs.jmedchem.8b01945.

57. Li, Y., J. Yang, A. Aguilar, D. McEachern, S. Przybranowski, L. Liu, et al. (2019). Discovery of MD-224 as a First-in-Class, Highly Potent, and Efficacious Proteolysis Targeting Chimera Murine Double Minute 2 Degrader Capable of Achieving Complete and Durable Tumor Regression*.* *J Med Chem* 62, 448-466. doi: 10.1021/acs.jmedchem.8b00909.

58. Vollmer, S., D. Cunoosamy, H. Lv, H. Feng, X. Li, Z. Nan, et al. (2020). Design, Synthesis, and Biological Evaluation of MEK PROTACs*.* *J Med Chem* 63, 157-162. doi: 10.1021/acs.jmedchem.9b00810.

59. Wei, J., J. Hu, L. Wang, L. Xie, M.S. Jin, X. Chen, et al. (2019). Discovery of a First-in-Class Mitogen-Activated Protein Kinase Kinase 1/2 Degrader*.* *J Med Chem* 62, 10897-10911. doi: 10.1021/acs.jmedchem.9b01528.

60. Wang, S., L. Han, J. Han, P. Li, Q. Ding, Q.J. Zhang, et al. (2019). Uncoupling of PARP1 trapping and inhibition using selective PARP1 degradation*.* *Nat Chem Biol* 15, 1223-1231. doi: 10.1038/s41589-019-0379-2.

61. Zhao, Q., T. Lan, S. Su, and Y. Rao (2019). Induction of apoptosis in MDA-MB-231 breast cancer cells by a PARP1-targeting PROTAC small molecule*.* *Chem Commun (Camb)* 55, 369-372. doi: 10.1039/c8cc07813k.

62. Bassi, Z.I., M.C. Fillmore, A.H. Miah, T.D. Chapman, C. Maller, E.J. Roberts, et al. (2018). Modulating PCAF/GCN5 Immune Cell Function through a PROTAC Approach*.* *ACS Chem Biol* 13, 2862-2867. doi: 10.1021/acschembio.8b00705.

63. Potjewyd, F., A.W. Turner, J. Beri, J.M. Rectenwald, J.L. Norris-Drouin, S.H. Cholensky, et al. (2020). Degradation of Polycomb Repressive Complex 2 with an EED-Targeted Bivalent Chemical Degrader*.* *Cell chemical biology* 27, 47-56 e15. doi: 10.1016/j.chembiol.2019.11.006.

64. Smith, B.E., S.L. Wang, S. Jaime-Figueroa, A. Harbin, J. Wang, B.D. Hamman, et al. (2019). Differential PROTAC substrate specificity dictated by orientation of recruited E3 ligase*.* *Nat Commun* 10, 131. doi: 10.1038/s41467-018-08027-7.

65. Li, W., C. Gao, L. Zhao, Z. Yuan, Y. Chen, and Y. Jiang (2018). Phthalimide conjugations for the degradation of oncogenic PI3K*.* *Eur J Med Chem* 151, 237-247. doi: 10.1016/j.ejmech.2018.03.066.

66. Mares, A., A.H. Miah, I.E.D. Smith, M. Rackham, A.R. Thawani, J. Cryan, et al. (2020). Extended pharmacodynamic responses observed upon PROTAC-mediated degradation of RIPK2*.* *Commun Biol* 3, 140. doi: 10.1038/s42003-020-0868-6.

67. Burslem, G.M., B.E. Smith, A.C. Lai, S. Jaime-Figueroa, D.C. McQuaid, D.P. Bondeson, et al. (2018). The Advantages of Targeted Protein Degradation Over Inhibition: An RTK Case Study*.* *Cell Chem Biol* 25, 67-77 e3. doi: S2451-9456(17)30353-7 [pii]

10.1016/j.chembiol.2017.09.009.

68. Tovell, H., A. Testa, H. Zhou, N. Shpiro, C. Crafter, A. Ciulli, et al. (2019). Design and Characterization of SGK3-PROTAC1, an Isoform Specific SGK3 Kinase PROTAC Degrader*.* *ACS Chem Biol* 14, 2024-2034. doi: 10.1021/acschembio.9b00505.

69. Wang, M., J. Lu, C.Y. Yang, and S. Wang (2020). Discovery of SHP2-D26 as a First, Potent, and Effective PROTAC Degrader of SHP2 Protein*.* *J Med Chem* 63, 7510-7528. doi: 10.1021/acs.jmedchem.0c00471.

70. Schiedel, M., D. Herp, S. Hammelmann, S. Swyter, A. Lehotzky, D. Robaa, et al. (2018). Chemically Induced Degradation of Sirtuin 2 (Sirt2) by a Proteolysis Targeting Chimera (PROTAC) Based on Sirtuin Rearranging Ligands (SirReals)*.* *J Med Chem* 61, 482-491. doi: 10.1021/acs.jmedchem.6b01872.

71. Wang, X., S. Feng, J. Fan, X. Li, Q. Wen, and N. Luo (2016). New strategy for renal fibrosis: Targeting Smad3 proteins for ubiquitination and degradation*.* *Biochem Pharmacol* 116, 200-9. doi: 10.1016/j.bcp.2016.07.017.

72. Farnaby, W., M. Koegl, M.J. Roy, C. Whitworth, E. Diers, N. Trainor, et al. (2019). BAF complex vulnerabilities in cancer demonstrated via structure-based PROTAC design*.* *Nature chemical biology* 15, 672-680. doi: 10.1038/s41589-019-0294-6.

73. Zhou, H., L. Bai, R. Xu, Y. Zhao, J. Chen, D. McEachern, et al. (2019). Structure-Based Discovery of SD-36 as a Potent, Selective, and Efficacious PROTAC Degrader of STAT3 Protein*.* *J Med Chem* 62, 11280-11300. doi: 10.1021/acs.jmedchem.9b01530.

74. Bai, L., H. Zhou, R. Xu, Y. Zhao, K. Chinnaswamy, D. McEachern, et al. (2019). A Potent and Selective Small-Molecule Degrader of STAT3 Achieves Complete Tumor Regression In Vivo*.* *Cancer Cell* 36, 498-511 e17. doi: 10.1016/j.ccell.2019.10.002.

75. Gechijian, L.N., D.L. Buckley, M.A. Lawlor, J.M. Reyes, J. Paulk, C.J. Ott, et al. (2018). Functional TRIM24 degrader via conjugation of ineffectual bromodomain and VHL ligands*.* *Nat Chem Biol* 14, 405-412. doi: 10.1038/s41589-018-0010-y.

76. Li, Z., B.J. Pinch, C.M. Olson, K.A. Donovan, R.P. Nowak, C.E. Mills, et al. (2020). Development and Characterization of a Wee1 Kinase Degrader*.* *Cell chemical biology* 27, 57-65 e9. doi: 10.1016/j.chembiol.2019.10.013.
